# The impact of thyroid disorders on the gut microbiome: emerging mechanisms and clinical relevance

**DOI:** 10.20945/2359-4292-2025-0075

**Published:** 2025-11-24

**Authors:** Isabela Busto Silva, Manuel Puig-Domingo

**Affiliations:** 1 Serviço de Endocrinologia e Metabologia do Hospital de Clínicas da Universidade Federal do Paraná, Curitiba, PR, Brasil; 2 Department of Endocrinology and Nutrition, Germans Trias Hospital and Research Institute, Universitat Autònoma de Barcelona, Barcelona, Spain

**Keywords:** Gastrointestinal microbiome, thyroid diseases, fatty acids, volatile

## Abstract

The thyroid-gut axis represents a dynamic interaction between the intestinal microbiota and thyroid function, with growing evidence linking gut dysbiosis to thyroid diseases. The gut microbiome, comprising over 100 trillion microorganisms, influences immune modulation, iodine metabolism, and thyroid hormone regulation. Short-chain fatty acids, produced by beneficial gut bacteria, support immune homeostasis and thyroid function, while pathogenic bacteria and lipopolysaccharides trigger inflammatory pathways that impair thyroid activity. Alterations in gut microbiota composition have been associated with autoimmune thyroid diseases, including Hashimoto’s thyroiditis and Graves’ disease. Dysbiosis increases intestinal permeability, antigen exposure, and immune activation, exacerbating thyroid autoimmunity. A reduction in short-chain fatty acids-producing bacteria weakens immune tolerance, promoting inflammatory cytokine release and autoantibody production. Recent studies highlight microbial metabolites such as tryptophan derivatives and their role in immune regulation. Gut dysbiosis is also implicated in thyroid nodules and cancer. Decreased butyrate-producing bacteria and increased inflammatory bacterial taxa have been observed in thyroid malignancies. Microbiota influence iodine and selenium bioavailability, essential for thyroid hormone synthesis, and modulate sodium-iodide symporter expression, affecting thyroid cancer response to radioactive iodine therapy. Microbiome-targeted interventions, including probiotics, prebiotics, dietary modifications, and fecal microbiota transplantation, may restore microbial balance, enhance immune regulation, and improve thyroid treatments. This review synthesizes our current understanding of the thyroid-gut axis, indicating that the intestinal microbiota and its metabolites may act directly or indirectly on the thyroid gland, highlighting potential clinical implications and paving the way for therapeutic strategies targeting the intestinal microbiota.

## INTRODUCTION

The alimentary tract creates a connection between the external environment and the body, and within it, there is a complex polymicrobial ecology that exerts an important influence on health and disease (^1^). A microbiome is a collection of microorganisms that plays a crucial role in immune development, so that any alteration in its homeostasis (dysbiosis) can cause and aggravate several diseases (^2^). It has individual characteristics; thus, it can be said that it confers individual signatures that may vary according to personal and environmental specific conditions applying for a given individual.

The association between alterations in the intestinal microbiota and thyroid diseases has gained prominence in recent years, due to the belief that it may play a fundamental role in the development and/or progression of thyroid diseases (^3^). This, in turn, encompasses a range of disorders, including autoimmune diseases and cancer, which not only impact metabolic regulation but also have significant implications for general health and well-being (^4^). In view of this, the interaction between the thyroid gland and the gut has emerged as an area of substantial interest in endocri nology (^5^), becoming a growing field of study, with research revealing the complex connection between the microbiota and the endocrine system (^4^).

This review explores recent advances in the gut microbiome’s role in thyroid diseases, focusing on its influence on autoimmune conditions like Hashimoto’s thyroiditis (HT) and Graves’ disease (GD), as well as its broader impact on non-autoimmune disorders, including thyroid nodules and cancer. By combining current knowledge on this topic, we aimed to enhance the understanding of the thyroid-gut axis, providing a theoretical basis for future studies and a new perspective for the potential implementation of microecological treatment strategies for thyroid diseases.

## THYROID-GUT AXIS

It has been estimated that the microbes in our bodies collectively comprise up to 100 trillion cells, most of which reside in the intestine. This observation suggests that they may encode one hundred times more genes than our own genome. Regarding the intestinal microbiota, it appears to be a tapestry of approximately 1,000 distinct bacterial species (^6^). Taxonomically, the phyla Firmicutes and Bacteroides predominate, constituting 70% to 75% of the gut microbiome (^3^). A high degree of gut microbiome diversity is crucial for maintaining overall health, as it supports immune function, nutrient absorption, and metabolic balance, and is strongly associated with reduced risks of chronic diseases and improved wellbeing. Several factors influence the composition of the microbiota, with four main components being highlighted: intrinsic factors related to the microbiome, such as composition, age dependency, and the presence of diseases; environmental factors, including local environment and vertical transmission; lifestyle factors, encompassing diet, medication usage, culture, and physical activity; and host genetics, involving body mass index, adipose tissue body topographical distribution, adaptive and innate immunity, and sex (^7^).

Beneficial bacteria from the phyla *Bifidobacterium* and *Lactobacillus* produce short-chain fatty acids (SCFAs) that are crucial for immunity regulation but also for the maintenance of thyroid hormone balance, while preserving the integrity of the intestinal barrier (^8^). In contrast, harmful bacteria, such as Bacteroides, Proteobacteria, and Actinobacteria, disrupt the balance of SCFAs by decreasing their production and promoting inflammation, impacting thyroid function. **Table 1** provides a concise overview of this influence on thyroid diseases.

**Table 1:** Comparison of intestinal microbiome patterns in individuals without thyroid disease, those with autoimmune thyroid disorders, and those with thyroid nodules and thyroid cancer

Parameter/bacterial group	Healthy individuals	Autoimmune thyroid diseases	Thyroid nodule	Thyroid cancer
Global diversity	High species diversity	Reduced diversity compared to healthy controls	Moderate reduction in diversity, but with variability	Discordant data: decreased diversity, possibly similar or even more pronounced than in autoimmune disease (^28^-^31^)
*Firmicutes: Bacteroidetes*	Relatively balanced	Possible dysregulation in the *Firmicutes: Bacteroidetes* ratio (increased *Bacteroidetes* and/or decreased *Firmicutes*)	Scarce data	Variable pattern. Increase in *Bacteroidetes* and decrease in *Firmicutes*
Short-chain fatty acid production	Normal	Reduction	Slightly reduced (few conclusive studies)	Generally reduced

There is a positive correlation between butyrate concentration, a SCFA, and the number of regulatory T cells (Tregs), which are key mediators of immune tolerance, as well as with lower concentrations of pro-inflammatory Th-17 cells. This is supported by observations that germ-free (gf) mice show impaired immune cell maturation due to the lack of microbial stimulation to the immune system (^9^). Treg cells, characterized by the expression of CD4, CD25, and the forkhead box P3 (Foxp3) transcription factor, are reported to aid in maintaining immune self-tolerance and suppressing autoreactive T and B cells through cell-to-cell contact or the secretion of regulatory cytokines such as interleukin-10 (IL-10) and transforming growth factor-β (TGF-β). Additionally, a reduction in propionic acid also contributes to the imbalance between Tregs and Th17 cells (^3^).

The thyroid gland requires iodine as an essen tial micronutrient for the synthesis of thyroid hormones. Absorption of this mineral from the gastrointestinal tract and its transfer to the thyroid gland constitutes the main pathway for iodine uptake in humans, but also selenium absorption is modulated by gut microbiota (^9^). In this context, intestinal microorganisms play a crucial role in the regulation of iodine metabolism in different manners. Thus, LPS and SCFAs released by the gut microbiota influences thyroid iodine metabolism by altering the expression and activity of the sodium-iodine symporter (NIS), as speculated in current research (^10^). But LPS also modulates thyroid homeostasis through a dual action on thyroid-stimulating hormone (TSH): by increasing gene expression of thyroglobulin (Tg) and NIS through the activation of TSH as demonstrated in a rat thyroid cell line (FRTL-5) (^11^) during infection, LPS or LPS-induced proinflammatory cytokines exert a direct action to stimulate the synthesis of deiodinase type II (D2) in the hypothalamus and anterior pituitary, thus facilitating the conversion of thyroxine (T4) to triiodothyronine (T3), which inhibits the production of TRH at the hypothalamus as well as TSH at the pituitary gland. D2 activity in the hypothalamus is linked to metabolic regulation, particularly in adaptive thermogenesis. By increasing local T3 levels, D2 influences energy expenditure and metabolic rate, which are critical for maintaining homeostasis. This activation of D2 has an important role in mediating the adaptation to central hypothyroidism that can occur during infection (^12^).

The intestinal microbiota plays a significant role in modulating thyroid function through various microbial mechanisms. Dysbiosis, characterized by an imbalance in microbial composition, leads to the deterioration of the intestinal barrier, increasing its permeability. This allows antigens to enter the circulation and activate the immune system, provoking the release of pro-inflammatory cytokines such as tumor necrosis factor alpha (TNF-α) and interleukin-6 (IL-6) (^13^). This activation can trigger a cascade of inflammatory responses which, if persistent, may culminate in chronic inflammation. Chronic inflammation, in turn, can induce cellular stress in the thyroid gland, activating stress pathways and resulting in the production of reactive oxygen species (ROS). This cellular stress may cause DNA damage and provoke changes in thyroid follicular cells (^3^). Furthermore, the inflammation and immune activation associated with increased intestinal permeability can impact the production and regulation of thyroid hormones, disrupting the normal feedback loop of the hypothalamic-pituitary-thyroid axis (H-P-T) and leading to hormonal imbalances (^14^).

Furthermore, SCFAs play a critical role in regulating the expression of the NIS, particularly butyric acid. It acts by inhibiting histone deacetylase, which in turn promotes the reexpression of NIS in thyroid cancer cells, leading to redifferentiation and enhanced iodine uptake. Given this, it can be interpreted that modulating SCFA levels may increase the sensitivity of tumor cells to radioactive iodine by promoting histone acetylation and enhancing NIS expression. However, further investigation is necessary (^10^).

Therefore, it is understood that the diversity and composition of the microbiome can also affect the bioavailability of iodine and selenium. Furthermore, it has been observed that in inflammatory bowel disease (IBD), there is a reduction in the diversity of the intestinal microbiota and a lower abundance of *Firmicutes* and *Bacteroidetes*, making poor iodine absorption a common consequence of IBD, and vice versa, suggesting a reciprocal relationship (^9^). **Table 2** summarizes the key points of these elements and their relationships with the gut microbiome in the context of thyroid diseases.

**Table 2:** Interaction between the elements and nutrients involved in thyroid diseases and the gut microbiome

Element/nutrient	Mechanism of action	Relationship with the gut microbiome
Sodium/iodide symporter	Transports iodine into thyroid cells along with sodium, a process dependent on concentration gradients	By influencing iodine absorption, gut dysbiosis can impair its bioavailability, impacting NIS function. Alterations in the microbiome can directly affect the expression of NIS in the thyroid
Iodine	Iodine, in the thyroid, is organified and incorporated into the synthesis of thyroid hormones	The gut microbiome can affect iodine absorption. It also influences the intestinal microbiota through its effect on the immune system
Selenium	Cofactor in enzymes such as deiodinase, responsible for the conversion of T4 to T3 Immunomodulator	The gut microbiome can influence selenium absorption, and its deficiency may exacerbate autoimmune thyroid disorders, as it has antioxidant and anti-inflammatory properties

NIS: sodium-iodide symporter.

**Figure 1:**
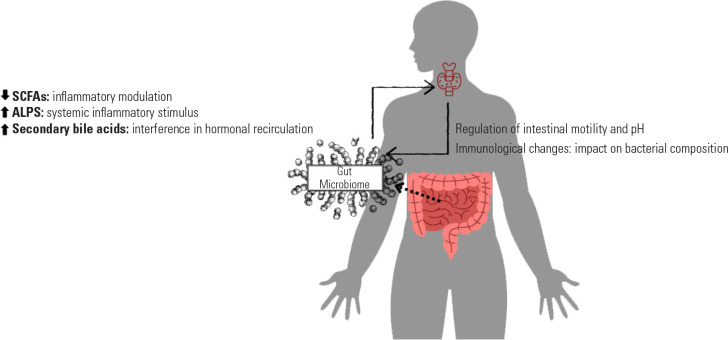
Relationships between the thyroid gland and the gut microbiota.

SCFA: short chain fatty acid; LPS: lipopolysacharides.

An additional point of fundamental relevance is the fact that the intestine plays another vital role as host to 70% of the body’s immune tissue, called gut-associated lymphoid tissue (GALT) (^15^). Gutassociated lymphoid tissue stores immune cells, such as T and B lymphocytes, which attack and produce antibodies against antigens. This supports the idea that a healthy gut microbiota can influence the immune system and has a significant impact on thyroid function (^3^). **Figure 1** provides a visual synthesis of the mechanisms by which alterations in the gut microbiome can exert effects on the thyroid gland, and vice versa.

## IMPACT OF MICROBIOTA ON AUTOIMMUNE THYROID DISEASES

Autoimmune thyroid disease (AITD) is the most common thyroid disorder, its two extremes being HT and GD (^16^). Some studies have reported its connection with the intestinal microbiota, however, the mechanisms of the complex intestine-thyroid axis have not yet been fully elucidated (^17^,^18^). In any case, it is speculated that intestinal microbiota dysbiosis in these diseases occurs through different mechanisms, such as bacterial overgrowth, overactivation of the inflammasome, increased intestinal permeability, alteration of microbiota metabolites, and immune homeostasis (^13^). In short, the changes observed indicate a potential breakdown in metabolic pathways and immunological balance, which may be relevant to the pathogenesis of AITD. These findings emphasize the significant role of gut microbiota in the development and progression of these pathological conditions (^19^).

Short-chain fatty acids, through their interaction with G protein-coupled receptors on leukocytes and intestinal epithelial cells, can inhibit AITD (^20^). In individuals with autoimmune primary hypothyroidism, a decrease in SCFA-producing bacteria leads to SCFAs decreased levels, exacerbating damage to the intestinal barrier and increasing serum LPS levels, which in turn can activate the immune system triggering the release of inflammatory mediators (^21^). Short-chain fatty acids reduction influences Tregs contributing to a Th1/Th2 imbalance, thus increasing the production of pro-inflammatory cytokines, such as interferongamma (IFN-γ) and TNF-α (^22^). The enhancement of this kind of immune response contributes to the breakdown of immunological tolerance and the gen eration of autoantibodies (^3^).

A significantly reduced proportion of circulating CD4 + Foxp3 + Treg cells has been observed in patients with recently diagnosed GD. This decrease in Treg cells may be partially explained by the increased apoptosis of these cells, as a higher number of apoptotic cells has been detected in the thyroids of patients with AITD. Furthermore, the proportion of Treg cells was inversely correlated with thyroid-stimulating antibody (TSAb) activity, suggesting that the deficiency of Treg cells in patients with GD may compromise their ability to inhibit the production of autoantibodies by B cells. Additionally, there is an increase in Th17 cells and elevated levels of IL-17 mRNA expression. Thus, it is proposed that the imbalance between Th17 and Tregs plays a crucial role in the pathogenesis of GD (^23^).

Advances in cellular immunology have ushered in a new era in exploring the mechanisms of diseases related to the immune system. It has been demonstrated that Th17 lymphocytes and their transcription factor are elevated in patients with AITDs; however, this increase is significantly more pronounced in patients with HT compared to those with GD. Therefore, it is speculated that Th17 plays a critical role in the pathogenesis of HT. Interestingly, unlike in GD, the differences in Tregs and their transcription factor were not significant between patients with Graves’ orbitopathy (GO) and healthy controls. Thus, it is suggested that Th17 has a critical influence on the development of GO, while Tregs primarily participate in the pathogenesis of GD. Ultimately, a disruption of the Th17/Treg balance occurs in AITDs (^24^).

Furthermore, a Mendelian randomization study, aiming to explore causality using statistics from several genome-wide association studies (GWASs) of gut microbes and fecal/circulating metabolites to assess potential causal associations of gut microbes and metabolites with AITD, highlighted the role of gut flora-related metabolites in the circulation, such as tryptophan, whose derivatives can interact with transcription factors such as nuclear factor-kappa B (NF-κB), estrogen receptor, and are capable of activating regulatory feedback loops highly relevant to immunoregulation. Also, circulating 3-indole glyoxylic acid in plasma showed a significant pathogenic role in AITD (^16^).

In turn, a meta-analysis (^13^) reported that the ratio of Firmicutes and Bacteroidetes of patients with AITD showed a lower level than their healthy counterparts. A random-effect model indicates that these patients have a significant increase in pathogenic bacteria together with a decreased proportion of beneficial bacteria, such as *Lactobacillus* and *Bifidobacterium*, these latter having anti-inflammatory effects. Another hypothesis on the relationship that the microbiota plays in the progress of thyroid autoimmunity is molecular mimicry, since the antigenic properties of proteins from certain intestinal bacteria can link TPO and TG, which are among the most relevant targets in the development of these diseases. It is hypothesized that genetic predisposition to AITD and/or intestinal epithelium penetration by α-1,6-glucan are required for triggering/acceleration or prevention/abrogation of AITD by bifidobacteria through molecular mimicry mechanism (^25^).

A field of continuous interest and progress in recent years is GO. Recently, a study (^26^) suggested that a possible increase in intestinal permeability is an aggravating factor for orbital inflammation and, therefore, for the differentiation of myofibroblasts and fibrosis. In view of this, intestinal permeability was assessed by measuring serum lipopolysaccharide-binding protein (LBP), zonulin, TLR5, and TLR9 ligands, and biopsies from the orbital tissue were studied. It was found that GO patients had significantly higher serum LBP concentrations than healthy controls, as well as circulating zonulin, TLR5, and TLR9 ligands, inferring that increased intestinal permeability was accompanied by enhanced expression of genes involved in immune cell infiltration and encoding key proteins for immune cell adhesion at the orbital tissue. Macrophage influx was positively linked to the extent of T cell influx and fibroblast activation within GO-affected orbital tissues. Equally important, serum LBP levels significantly correlated with the abundance of specific intestinal *Gram*negative bacteria, linking the gut to local orbital inflammation.

## IMPACT OF MICROBIOTA ON THYROID NODULES AND CANCER

The development of thyroid nodules is multifactorial, involving genetic predisposition, environmental exposures, and hormonal factors. It is already known that the risk of thyroid cancer arising within a nodule is influenced by various factors; however, there is growing interest in the influence of the intestinal microbiome in the context of thyroid nodules (^3^).

Studies show a significant increase in the relative abundance of *Neisseria* and *Streptococcus* and a notable decrease in microbes such as *Butyricimonas* and *Lactobacillus* in patients with thyroid nodules (^27^). Furthermore, the reduction in the metabolic pathways responsible for producing the SCFA butyrate, along with an upregulation of amino acid biosynthesis pathways, as a consequence of the disruption of intestinal microbial metabolism that may stimulate nodule development (^28^). Some studies have suggested that LPS may exert a direct effect on thyroid cells, influencing their growth and function, and potentially leading to nodule formation through (^29^). Moreover, the impact of the intestinal microbiome on thyroid nodules generation and growth can be explained by its ability to modulate the production of cytokines such as IL-1β, IL-6, and TNF-α, which are known to influence the proliferation of thyroid cells (^30^).

Regarding thyroid cancer, the role of the intestinal microbiome in its pathophysiology is also an emerging area of research, especially considering that it is the most common endocrine malignancy, with globally increasing incidence in recent decades (^31^). There is no doubt that evidence points to a fundamental interaction between the intestinal microbiota and the thyroid gland; however, the literature presents some discrepancies concerning the nature of this interaction. While some studies have indicated that patients with thyroid cancer show higher intestinal microbiota richness and diversity compared to a control group of healthy individuals (^32^,^33^), other studies report a significant decrease (^34^,^35^). Despite these contradictory findings, more data seem to support a decline in the abundance of SCFA-producing bacteria in patients with thyroid cancer (^10^), suggesting that this reduction are associated to disease progression (^36^).

A recent Mendelian randomization study (^36^) - a novel statistical approach that allows researchers to assess causal relationships in potential exposure-outcome pathways - indicated that an increased abundance of *Butyrivibrio, Fusicatenibacter, Oscillospira, Ruminococcus* 2, and *Terrisporobacter* may contribute to a higher risk of thyroid cancer, whereas elevated levels of *Olsenella* and *Ruminococcaceae UCG-004* were associated with a reduced risk. In this way, the study offered genetic evidence supporting the existence of a gut-thyroid axis, emphasizing the strong correlation between the intestinal microbiota and thyroid cancer.

The complex interaction between the thyroid and the gut becomes even clearer when one recognizes that it is bidirectional, as the thyroid and/or its treatment can also affect the composition and activity of the intestinal microbiome. A pilot study (^37^) reported a significant decline in the *Firmicutes-to-Bacteroides* ratio following radioactive iodine (I131) therapy in patients who had undergone total thyroidectomy for papillary thyroid cancer. In addition, it was noted that *Dorea* (a butyric acid-producing genus) was significantly less abundant prior to treatment in patients who showed a lower response post-I131, suggesting its potential pathogenic importance and its predictive value (^37^,^38^).

## CLINICAL PERSPECTIVES

The integration of microbiome-based therapies with traditional treatments represents a paradigm shift in many fields and also in thyroid disease management. Personalized approaches, tailored to an individual’s gut microbiota composition, could potentially optimize therapeutic outcomes and minimize adverse effects. For example, dietary interventions aimed at restoring microbial balance, such as the elimination of gluten in patients with celiac disease or non-celiac wheat sensitivity, have shown promising results in alleviating thyroid dysfunction (^9^). Similarly, the use of synbiotics - combinations of probiotics and prebiotics - may offer synergistic benefits in modulating the gutthyroid axis (^39^).

As previously commented, the role of gut microbiota in thyroid cancer is an area of growing interest. Dysbiosis has been associated with an increased abundance of carcinogenic and inflammatory bac terial strains, which may contribute to tumorigenesis (^40^). For instance, certain bacterial taxa, such as *Ruminococcaceae UCG004* and *Streptococcaceae*, have been implicated in thyroid cancer progression through mechanisms involving immune modulation and metabolic pathway alterations, thus decreasing the gut content of these specific strains may potentially be of benefit for thyroid cancer patients. Additionally, gut microbiota can influence the efficacy of radioactive iodine therapy by modulating the expression of NIS, which facilitates iodine uptake in thyroid cancer cells; thus, microbiota-derived metabolites like SCFAs can enhance the sensitivity of tumor cells to radioactive iodine, potentially improving treatment outcomes.

The modulation of gut microbiota through probiotics, prebiotics, and fecal microbiota transplantation (FMT) has shown promise in managing thyroid disorders. Specific probiotic strains, such as *Lactiplantibacillus plantarum 299v* and *Bifidobacterium longum*, have been demonstrated to restore microbiota balance, improve intestinal barrier function, and reduce systemic inflammation (^3^). These probiotics can enhance the effects of conventional treatments, such as levothyroxine (L-T4) therapy for hypothyroidism, by optimizing absorption and stabilizing the required dosage of L-T4. However, other studies have suggested that probiotics, generally containing *Bifidobacterium* spp. and *Lactobacillus* spp., have not been able to alter susceptibility or improve hypothyroidism in patients with HT. Interactions between probiotics, L-T4 therapy and H-P-T were examined, leading to the conclusion that probiotics are unlikely to be beneficial in the treatment of patients with TH (^41^). This finding is particularly noteworthy given that significantly lower levels of *Lactobacillus* spp. have been identified in patients with thyroid nodules and thyroid cancer, suggesting a link between the absence of this genus and thyroid disease. Nonetheless, it is possible that the duration of treatment was too short to achieve a significant change in microbiota composition (^7^,^41^).

Fecal microbiota transplantation has emerged as a potential therapeutic option in GD. By recalibrating gut microbiota, FMT can influence neurotransmitter activity and trace element metabolism via the gut-brain and gut-thyroid axes, offering a holistic approach to disease management (^3^). However, while these interventions hold promise, further large-scale clinical trials are needed to establish standardized protocols and validate their efficacy across diverse patient populations.

The gut microbiota plays a crucial role in modulating selenium bioavailability, which is essential for the synthesis of selenoproteins, including glutathione peroxidases and iodothyronine deiodinases, enzymes that are critical for thyroid hormone metabolism (^39^). Dysbiosis in the gut microbiota has been shown to impair selenium absorption, leading to reduced selenoprotein activity, which in turn affects thyroid hormone conversion and increases susceptibility to thyroid dysfunction (^42^). Studies indicate that certain gut bacteria, such as *Lactobacillus* and *Bifidobacterium* species, can enhance selenium bioavailability, thereby protecting thyroid hormone homeostasis and reducing the risk of hypothyroidism (^43^). Reduced gut microbial diversity has been associated with altered selenium metabolism, leading to suboptimal thyroid function due to impaired conversion of T4 to the biologically active T3 hormone (^44^). Therefore, targeting dysbiosis for rebalancing the specific microbiome strains involved in selenium malabsorption may be crucial for preservation of thyroid function through maximizing selenium bioavailability in patients in which this mineral is given as therapeutic supplementation for AITD.

Future research should focus on elucidating the specific mechanisms underlying the thyroid-gut axis, including the role of microbial metabolites like SCFAs and bile acids in thyroid hormone metabolism. Advanced techniques, such as metagenomic sequencing and metabolomic profiling, can provide deeper insights into the complex interactions between gut microbiota and thyroid function. Additionally, longitudinal studies are needed to assess the long-term effects of microbiota-modulating interventions on thyroid health.

## CONCLUSIONS

The thyroid-gut axis highlights the profound impact of gut microbiota on thyroid function and disease. From autoimmune disorders to thyroid cancer, dysbiosis plays an important role in disease pathogenesis and progression. Therapeutic strategies targeting the gut microbiota, such as probiotics, fecal microbiota transplantation, and dietary interventions, offer promising avenues for improving patient outcomes. As research in this field continues to evolve, the integration of microbiome-based therapies into clinical practice holds the potential to revolutionize the management of thyroid diseases, paving the way for more personalized and effective treatments.

By leveraging the insights gained from the thyroidgut axis, clinicians can adopt a more holistic approach to thyroid care, addressing not only the gland itself but also the intricate microbial ecosystem that influences its function. This paradigm shift underscores the importance of considering the gut microbiota as a key player in thyroid health and disease, opening new frontiers in endocrinology and personalized medicine.

## Data Availability

datasets related to this article will be available upon request to the corresponding author.
